# Building the UK's industrial base in engineering biology

**DOI:** 10.1049/enb2.12016

**Published:** 2021-12-06

**Authors:** Richard I. Kitney

**Affiliations:** ^1^ UK National Centre for the Industrial Translation of Engineering Biology/Synthetic Biology (SynbiCITE) Imperial College London London UK; ^2^ Department of Bioengineering Imperial College London London UK

**Keywords:** bio‐economy, Engineering Biology, industrial translation, industry

## Abstract

The paper describes the strategy and components that have been put in place to build the UK's research and industrial base in Engineering Biology. The initial section of the paper provides a brief historical overview of the development of the field in the United Kingdom. This comprised, principally, a major report by the Royal Academy of Engineering and a strategic roadmap for synthetic biology, together with the establishment of six new synthetic biology research centres, a national centre for the industrial translation of synthetic biology and five biofoundries. The next section of the paper describes the UK government’s policy for the field. Important elements of the implementation of the policy comprises people, Infrastructure, Business Environment and place. In this context, a number of important areas are addressed—including industrial translation; building an expert workforce and nucleating, incubating and accelerating a new engineering biology industry in the United Kingdom. The final portion of the paper addresses the author's view of the way forward. This comprises placing the development of the field, both nationally and internationally, in the context of the development of the Bioeconomy and Climate Change. The final section of the text addresses a specific strategic approach and the implications for the United Kingdom in relation to the development of its industrial base in Engineering Biology.

## 1 BACKGROUND

Prior to 2008, there were several laboratories, principally at Cambridge and Imperial College that were engaged in Engineering Biology research^1^. In addition, teams from these universities had successfully taken part in iGEM (at that time, the MIT‐based International Genetically‐Engineered Machine competition for students [1]). (It is important to note that iGEM has been extremely internationally influential in attracting students into the field of Engineering Biology, many of whom have stayed in the field and some are now the CEOs of successful companies.) In 2008, the UK's funding agencies became sufficiently interested in Engineering Biology that it was incorporated into an Open Call for the Engineering and Physical Sciences Research Council (EPSRC) Science and Innovation Awards. Imperial College was successful in obtaining an award under the Call and established the first of the UK's Synthetic Biology centres (Centre for Synthetic Biology and Innovation, CSynBI at Imperial College).

At around this time The Royal Academy of Engineering were persuaded that Synthetic Biology was important to the future of engineering and in the future likely to be important in terms of the UK's economic growth. A decade later, Synthetic Biology or Engineering Biology, as it is now often called, was and is a key component of the UK Government's strategy for the development of the BioEconomy. (The terms Engineering Biology and Synthetic Biology are now often used interchangeably in the literature; hence, in this paper, the term Engineering Biology will be used throughout.) The Royal Academy of Engineering undertook an Inquiry, and the Report was published in May 2009 [2]. The report contained several important recommendations, which subsequently became the basis of the UK's future strategy for Engineering Biology. These were (in summary)

1. That the UK Government needed to develop a strategy to establish the country as an international leader in Engineering Biology. The involvement of industry in developing a strategy for Engineering Biology will ensure that research becomes progressively more directed as it becomes more applied. This will ensure a more rapid and successful translation of research into commercial applications
2. Number of academic centres dedicated to the subject are required. These centres should be located within leading universities that have internationally competitive research in engineering and physical sciences and biology. They must be truly multi‐disciplinary, with the ability to carry out world‐leading research. Wherever possible, the centres should be based in universities with existing activities in Engineering Biology, in order to maximise the UK’s capacity in the field at the lowest cost
3. It is essential that the centres should seek partnerships with the industry to ensure that projects of high national economic importance receive priority. This might mean developing and applying new techniques to the existing industry—for example the biotech industry as well as nurturing new and existing small and medium sized enterprises (SMEs)

In parallel with the report, the ‘so‐called’ Six Academies Meetings on Engineering Biology were organised by Johnson and Kitney [3]. These meetings comprised the National Academies of Engineering and Science in the United Kingdom, United States and China and were held in London, Shanghai and Washington between April 2011 and June 2012. The then Minister for Research and Universities, The Rt Hon David Willetts MP, attended the first of the workshops in London in April 2011 and became convinced of the importance of the field. This subsequently resulted in the organisation of a roundtable discussion chaired by the minister in November 2011 and the establishment of a working party to write a strategic roadmap for Engineering Biology. The report—A UK Roadmap for Engineering Biology—was published in July 2012 [4].

The main recommendations of the UK Roadmap incorporated the recommendations of The Royal Academy of Engineering Report and translated into the Government investing around £350 million around 2013/2014 in six new Engineering Biology research centres (the SBRCs) in Bristol, Cambridge, Edinburgh, Manchester, Nottingham and Warwick together with the UK's National Centre for the Industrial Translation of Engineering Biology/Synthetic Biology (SynbiCITE). [5]

An outline description of the research emphasis of each of these centres and the existing centre at Imperial College is given below.

- OpenPlant [6] is: (i) developing new tools and methods for plant engineering biology/synthetic biology, (ii) providing mechanisms for open sharing of standardised resources, (iii) applying these tools to world‐leading projects in trait development, and (iv) facilitating interdisciplinary exchange, outreach and international development
- BrisSynBio [7] is a multi‐disciplinary research centre that focusses on the biomolecular design and engineering aspects of Engineering Biology/Synthetic Biology
- SYNBIOCHEM [8] is a UK/European centre of excellence for Engineering Biology/Synthetic Biology of fine and speciality chemicals production (including new products and intermediates for drug development, agrochemical, and new materials for sustainable bio manufacturing)
- SynBio Nottingham [9] concentrates on engineering bacteria to make industrially useful products from C1‐feedstocks including carbon monoxide and greenhouse gases: carbon dioxide and methane.
- Warwick (WISB) [10] combines the principles of bioscience, engineering, computer science and physical science in theoretical and experimental Engineering Biology/Synthetic Biology research. WISB is developing next‐generation Engineering Biology/Synthetic Biology tools and systems, biosynthetic pathways, synthetic communities of microbes, and plant–microbe interactions
- Edinburgh’s Mammalian Engineering Biology/Synthetic Biology Centre [11] has an ambitious plan to build expertise in cell engineering tool generation, whole‐cell modelling, computer‐assisted design and construction of DNA and high‐throughput phenotyping to enable Engineering Biology/Synthetic Biology in mammalian systems for multiple applications.
- Imperial College’s Engineering Biology/Synthetic Biology research centre (Centre for Engineering Biology/Synthetic Biology and Innovation—CSynBI; now IC‐CSynB [12]) was established under an EPSRC Science and Innovation Award in the spring of 2009. The research strategy for the centre continues to be one of the developing platform technology for Engineering Biology/Synthetic Biology that can be applied across a wide range of applications.

Five UK Biofoundries at Earlham [13], Edinburgh [14], Imperial [15], Liverpool [16], and Manchester [17] were funded in 2014.

In the autumn of 2012, the Government established the Synthetic Biology Leadership Council (the SBLC)^2^ to review the strategy for Synthetic Biology on a regular basis. This resulted in the writing and publication of an update to the original UK Roadmap in February 2016 [18]. The new roadmap made five recommendations:

1. Accelerate industrialisation and commercialisation. By promoting investment in, and translation of, empowering Biodesign technologies and assets to drive growth in the BioEconomy.
2. Maximise the capability of the innovation pipeline by continuing to research and develop platform technologies that will improve manufacturing efficiencies and unlock future opportunities.
3. Build an expert workforce. By distilling the skills required for BioDesign and implementing them through education and training.
4. Develop a supportive business environment. By promoting strong and integrated governance, a proportionate regulatory system, excellent stakeholder relationships and responsible innovation (RI).
5. Build value from national and international partnerships. By fully integrating the UK Engineering Biology community to position UK research, industry and policy makers as partners of choice for international collaboration.

These recommendations became the basis of much of the subsequent strategy for Engineering Biology in the United Kingdom.

## 2 GOVERNMENT POLICY

In December 2018 the UK Government published a report entitled ‘Growing the BioEconomy’ [19]. In the report, there is a statement that ‘we aim to create the right supportive environment in the United Kingdom to help double the size of the impact of the BioEconomy from £220 billion in 2016 to £440 billion by 2030’. The field of Engineering Biology is seen as an important driver of this growth and also in its relationship to industrial biotechnology. Both are seen as ‘game changing fields’. Specific areas of development relating to the BioEconomy as defined by the Department for Business, Energy and Industrial Strategy (BEIS) are namely, People, Infrastructure, Business Environment and Place.

### 2.1 People

In relation to Engineering Biology, under this heading comes the objective of building an expert workforce. Within the 2018 report, the Government emphasises the need for increased STEM skills (science, technology, engineering and mathematics) across the board. Specifically, there are now a number of courses within universities throughout the United Kingdom covering Engineering Biology (mainly in terms of modules). However, from an educational point of view, the primary driver is still PhD programmes. There are currently two centres for doctoral training. The SynBio Centre (comprising the universities of Bristol, Oxford and Warwick) and the Imperial Centre (IC‐CSynB) (comprising the universities of Imperial College, Manchester and the University College, London). It is perhaps important to note that many of the students undertaking PhD's have been members of iGEM teams in the past. A fairly common route is for such students, upon completing their project, to form a company on the basis of their PhD area.

### 2.2 Infrastructure

In terms of the current infrastructure for Engineering Biology, this currently comprises the seven basic research centres in Bristol (BrisSynBio), Cambridge/John Innes (OpenPlant), Edinburgh (Centre for Mammalian Engineering Biology), Imperial (IC‐CSynB), Manchester (Synbiochem), Nottingham (SynBio), and Warwick (WISB). There are five Biofoundries in Edinburgh (the Edinburgh Genome Foundry), the Earlham Institute, Imperial College (the London Biofoundry), Liverpool (GeneMill), and Manchester (Synbiochem). SynbiCITE based at Imperial College is the UK's National Industrial Translation Centre for Engineering Biology. Other infrastructure facilities comprise the GSK bio facility, UNIT DX Science Incubator in Bristol, the Materials Innovation Factory, UK Bio manufacturing Research Hub, IBioIC and the CPI.

### 2.3 Business environment

The UK Government aims to foster an environment that allows the BioEconomy to thrive. This is seen as being achieved through ‘the creation of new jobs, increased investment and the delivery of values right across the United Kingdom’. In addition, the objective is to secure global investment in the sector and export deals. In the Growing the BioEconomy Report, a case study is cited, which is the Bio‐preferred Program of the US Department of Agriculture. This illustrates how federal procurement can stimulate the marketplace. One factor that is seen as important is the use of ‘long‐tail’ public funding to reduce financial risk within the field of Engineering Biology for private investors (the DARPA program in United States is a good example of this approach).

### 2.4 Place

The UK Government strategy, as defined within the Growing the BioEconomy Report, is one of building the BioEconomy from its roots in rural and coastal communities, industrial clusters and knowledge centres in all parts of the United Kingdom. As can be seen from the previous sections, the infrastructure for Engineering Biology matches this concept. The benefits of the BioEconomy and its development need to be across the whole of the United Kingdom and not, simply, in the traditional areas of growth around London and the South‐East of England. The strength of the BioEconomy is seen as coming from its decentralised nature.

## 3 THE NATIONAL INDUSTRIAL TRANSLATION CENTRE FOR ENGINEERING BIOLOGY/SYNTHETIC BIOLOGY (SynbiCITE)

SynbiCITE's overall vision is to work within the UK's Engineering Biology innovation and academic ecosystem to create a highly interconnected UK innovation cluster. Although the field of Engineering Biology is rapidly expanding in the United Kingdom and globally, the original strategy of industrial translation—principally, developing start‐up companies and supporting SMEs—remains valid. However, in the United Kingdom three major elements have changed since 2013: namely, the creation of the Engineering Biology Research Centres (SBRC's); the publication of various government reports (outlined above); and the experience gained in establishing and operating SynbiCITE. The overriding objective for SynbiCITE remains one of the providing support in three ways: (a) by providing and channelling scientific and technical expertise; (b) by acting as a conduit for funding and investing—particularly private sector funding; and (c) by providing business education and training. These are seen as key components in the industrial translation of the UK's science base. SynbiCITE's strategy is aligned with the key objectives of accelerating industrial translation and commercialisation through creating and supporting companies; building an expert workforce; providing business education and a supportive business environment; and building values through national and international partnerships.

Again, in relation to the SynbiCITE strategy, a key application area for Engineering Biology is industrial translation (i.e. the development and integration of a process that starts with BioDesign and ends with advanced biomanufacturing). To scale industrial translation requires technical standards, metrology, and higher levels of reliability and reproducibility. Consequently, an important change in SynbiCITE's strategy, since its establishment in 2013, has been the conceptualisation and development of DNA foundries (of which the London Biofoundry is a good example). The concept and development of Bio foundries represents a paradigm shift in the ability to undertake much more effective BioDesign‐led projects, leading to much better reliability and reproducibility, in terms of industrial translation. The need to develop much better metrology techniques has been the driver for the development of the collaboration between SynbiCITE and the UK's National Physical Laboratory [20]. This has resulted in the establishment of a joint Centre of Excellence for Metrology and Standards for Engineering Biology industry through funding from BEIS^3^ [21].

In practical terms, the work of SynbiCITE is divided into three hubs of activity:

- The Science, Engineering, Bio design and Applications Hub—design, proof of concept (PoC) projects, and development projects
- The Business and Outreach Hub—business education and training, and investor consortium, an industrial club, communications and PR
- The Facilities Hub—the London Bio foundry, analytics, design, automation development, project and company support

## 4 EDUCATION AND TRAINING AND EXPERT WORKFORCE

### 4.1 University‐based courses

There is an expanding number of university‐based courses now running in the United Kingdom (and in some cases that have been running for a decade). Examples are

**Edinburgh—**Postgraduate: PhD students; MSc in Synthetic and Systems Biology; MSc in Synthetic and Biotech.
**Imperial—**Undergraduate: Engineering Biology Modules that slot into more traditional undergraduate courses, for example, options as part of Bioengineering BEng and MEng. Postgraduate: MRes/PhD in Systems and Engineering Biology.
**Manchester and Nottingham:** Postgraduate—PhD students
**Oxford, Bristol and Warwick Centre for Doctoral Training:** Postgraduate—PhDs students

### 4.2 Retraining courses

There are a number of retraining courses that take place across the United Kingdom. Manchester is a good example. They run retraining courses, that is, continuing professional development‐type events where industry groups attend small workshops. The workshops comprise bespoke training, for example in the bio catalysis and chemicals biomanufacturing areas. These take a variety of formats.

### 4.3 iGEM

iGEM is important both from the educational and industrial standpoints. Participation in iGEM has resulted in some successful start‐ups being created (e.g. LabGenius, Puraffinity—previously called CustoMem). iGEM creates enthusiasm amongst students for Engineering Biology. There are many examples in the United Kingdom of students who have undertaken iGEM going on to do PhDs in Engineering Biology and, in some cases, to start successful companies.

### 4.4 SynbiCITE's Business Education and Training (undertaken across the United Kingdom)

This comprises two main activities—the 4‐Day MBA course and Lean LaunchPad/BioStart. The aim is to focus, principally, on the task of providing a supportive business environment for start‐ups and growing SMEs. ***The 4‐Day MBA*** is designed to provide a rapid introduction to the key elements of business practice that are needed to establish and grow a new company. (Here, the acronym stands for More Business Administration.) An important aspect of the course is that no prior knowledge is assumed. ***Lean LaunchPad/BioStart*** was originally developed within SynbiCITE to provide a customer‐facing course for start‐ups and SMEs in Engineering Biology. The aim of the course is that through extensive mentoring, by appropriate business experts, the teams develop an effective product/business strategy that can be funded by external investment/grants at the end of the course.

## 5 NUCLEATING, INCUBATING AND ACCELERATING A NEW INDUSTRY IN THE UNITED KINGDOM

Within the UK's Engineering Biology ecosystem, there are examples of start‐ups outside SynbiCITE; nevertheless, SynbiCITE is the vehicle that provides major support for company growth within the United Kingdom. To recapitulate, its mission is to promote the adoption and use of Engineering Biology by the industry. The primary objective is to accelerate and promote the commercial exploitation of Engineering Biology research from universities and other institutions throughout the United Kingdom. In this regard, SynbiCITE acts as a nucleating point for Engineering Biology companies and, particularly, start‐ups and SMEs. To put this in perspective, over the last 5 years there have been: around 80 significant business collaborations/interactions (i.e. PoC), Development Projects and other collaborative activity—foundry, analytics etc.); 40 industrial partners; collaboration with over 27 UK Universities; 25 PoC/development projects funded; and 418 people have attended the 4‐day MBA, Lean LaunchPad, BioStart and teach the teachers courses. The ‘Start‐up Survey’, produced by SynbiCITE [22] shows that United Kingdom has a growing and vibrant Engineering Biology start‐up and SME ecosystem. Examples of such companies are:

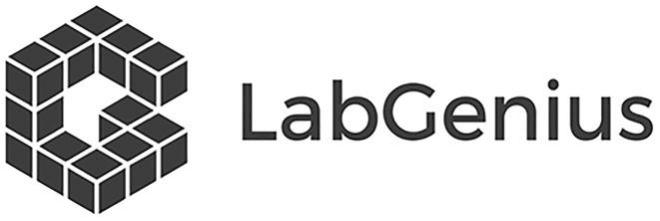

**Founder and CEO:** James Field
**Description:** LabGenius [23] develops next‐generation protein therapeutics using a machine learning‐driven evolution engine (EVA). The company uses robotic automation, synthetic biology and advanced machine learning to explore protein fitness landscapes and improve multiple drug properties, simultaneously. It is a privately‐owned company, backed by top‐tier venture capital funds, currently based in London. Private investment to date is £24.3 m

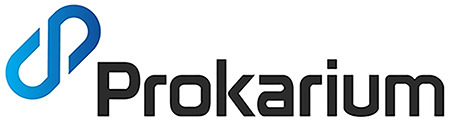

**Founder and Former CEO:** Ted Fjallman
**Description:** Prokarium [24] is pioneering the field of microbial immunotherapy. The company's pipeline is designed to unlock the next level of immuno‐oncology by building on the most recent advances in cancer immunology. Prokarium's lead programme is focussed on transforming the treatment paradigm in bladder cancer by orchestrating immune‐driven, long‐lasting antitumour effects. Private investment to date is £23.4 m

## 6 INVESTMENT

Various estimates place the British Government's investment in Engineering Biology at around £350 million, primarily in 2013/2014, to establish the new SBRCs, the Biofoundries and SynbiCITE. As part of its work SynbiCITE has a continuing review of private sector investment into companies working in the United Kingdom in Engineering Biology. From 2013 to 2020 there was a total investment of £2.19 billion into the sector, showing a steady increase in investment year‐on‐year (e.g. the figures for 2017 and 2018 are £262 m and £500 m, respectively). This represents a 6× multiplier on the public sector investment. Many analysts in the field believe that there is a need for a different type of funding model for small companies. In many large companies and, indeed, in the traditional VC investment community, Engineering Biology is still viewed as ‘high risk’. In the United States, for example, this problem has been recognised in several fields over the last 30 or 40 years. One solution, which has been highly effective, is to provide a ‘long‐tail’ of public funding over many years (e.g. the DARPA program) to ‘de‐risk’ the field to the point where the private sector feels comfortable about investing large amounts of money.

## 7 THE COMMITMENT TO RI

An important component of RI in the United Kingdom revolves around the use of the EPSRC ‘Area’ Framework [25]. Within the framework, RI is defined as ‘a process that seeks to promote creativity and opportunities for science and innovation that are socially desirable and undertaken in the public interest’. Across the United Kingdom RI is embedded for example in the 4‐Day MBA and the BioStart Accelerator Programme, collaborative project development with both universities and industry; and workshops and advice for start‐ups. PoC and DoP projects frequently include an RI fund/no fund testing element. There is also ongoing investor education as well as visits to start‐ups and SMEs. An important recent development is the embedding of RI across the new Future Bio Manufacturing Institute at Manchester. In addition, in relation to RI, the UK's commitment to developing suitable guidelines for industry is also reflected in BSI PAS 440:2020 ‘Responsible Innovation—Guide’. [26]

## 8 NATIONAL AND INTERNATIONAL STRATEGIC INITIATIVES RELATING TO PARTNERSHIP

Over the last few years, there have been two major additional initiatives by the UK Engineering Biology community. These are the development of the SynbiTECH series of conferences and the Global Biofoundries Alliance.

### 8.1 SynbiTECH

The SynbiTECH conferences [27] are primarily a showcase for the development of the Engineering Biology industry in the United Kingdom and internationally. Following a pilot in 2018, the 2019 SynbiTECH conference took place in June in the Queen Elizabeth II Conference Centre in Central London. The 2‐day conference attracted almost 400 attendees from 14 countries, with 76 speakers and 29 sponsors and exhibitors. There were over 3.5 million Tweets relating to the conference by delegates. General feedback from the conference was that delegates felt that, uniformly, there was excellent communication and connectivity with the whole of the Engineering Biology and industrial biotechnology communities.

### 8.2 The Global Biofoundries Alliance

The Global Biofoundries Alliance [28] was launched in May 2019, with the following objectives:

(a) To accelerate and enhance non‐commercial research in Engineering Biology
(b) To build a robust Engineering Biology industry and accelerate commercialisation of engineering/Engineering Biology and biomanufacturing process engineering with broad public benefits
(c) To promote and enable the beneficial use of automation and high‐throughput equipment including process scale‐up, computer‐aided design software, and other new workflows and tools in engineering/Engineering Biology.

### 8.3 International collaboration

Many British universities are engaged in international collaborative projects with leading universities and industries in a range of developed countries (e.g. the US, China, Singapore and Australia) as well as in developing countries, such as Kenya. In addition, it should be noted that the UK Engineering Biology Leadership Council responds formally to requests relating to Engineering Biology/Synthetic Biology‐related studies from the UN CBD (convention for BioDiversity) on behalf of the community.

## 9 INTERNATIONAL DEVELOPMENT

To ensure that the UK's strategies and policies remain relevant to shifting global developments, it is very important that the United Kingdom tracks Engineering Biology developments. For example, the United Kingdom should track the United States, China, Singapore and Australia not only in terms of developments within specific centres, (e.g. universities and industry) but also, in relation to government reports and roadmaps. By way of a specific example, there is significant collaboration with the Engineering Biology Research Consortium (EBRC) of the United States. This comprises several areas of work. For example, in relation to the development of the EBRC Roadmap and the development of the EBRC's international strategy forum for Engineering Biology [29].

### 9.1 The way forward

A number of countries have developed their own Engineering Biology roadmaps. What is interesting is how much emphasis is being placed on the development of a new technology to address climate change and to achieve economic and industrial sustainability. In the United States, the Biden Administration is addressing this issue seriously—for example, through a new Act entitled ‘Endless Frontier’. In the Act, the sponsors identify Synthetic Biology as one of a small number of key areas of technology that need to be supported by the US Government. Currently, in the Act there is an allocation of 100 billion USD, over 5 years, to back the key development areas—of which one is Synthetic Biology. It is interesting to note that in a recent survey, the issue of how nations incorporate the use of biotechnology and Engineering Biology in their Bioeconomy strategies is addressed. Of the 16 countries and organisations reviewed [30], only the United States and United Kingdom show any significant reliance on the use of Engineering Biology. Japan sees Engineering Biology as of some importance in this context and Germany, Norway and South Africa identified the limited use of Engineering Biology in developing their bio economies. What is striking is that the majority of countries and organisations in the survey are relying very heavily on sustainable resources, with only limited reliance on biotechnology—the exceptions being Japan and South Africa. In the case of United States and the United Kingdom, there is roughly equal reliance on sustainability, with the United States relying more heavily on Engineering Biology developments, as opposed to biotechnology, as compared to the United Kingdom [30].

Enabling the development of an advanced BioEconomy through public policy supporting Engineering Biology developments—through the use of automation AI and machine learning (e.g. as realised in Biofoundries) —is seen as being essential if high levels of reproducibility and reliability are to be achieved in biologically‐based industrial processes. This is not simply a matter of updating standard biological processes, but, rather, one of the interplay between BioDesign, based in software (so‐called bio CAD) and the use of high levels of automation [31]. The question is how realistic is the alternative economic model of bio‐based feedstocks (biomass) feeding through Engineering Biology to industrial processes and products to achieve a sustainable economy? In terms of lignocellulosic waste resources, there are significant amounts of wheat straw, corn stover, bagasse, rice straw and other grain straw distributed around many areas of the world. These resources are, for example, described in detail in a report by the KTN in the United Kingdom entitled from ‘Shale Gas to Biomass’ [32].

The second of the two major problems facing the world (the first being climate change/sustainability) relates to the coronavirus pandemic and the ability to deal with future pandemics. As with the development of the BioEconomy, a question which is now being asked is what role does Engineering Biology have to play in making the vaccines industry more sustainable? The question can be answered both generally and more specifically. In general terms, Engineering Biology can make the vaccines industry more sustainable in ways that translate into higher levels of reliability and sustainability. Again, through the use of BioDesign and the extensive use of automation, coupled to AI and machine learning [33]. An adjunct question, often posed, is how, specifically, can Engineering Biology accelerate and improve vaccine development and manufacturing? In the case of Moderna, with the support of the Boston‐based engineering biology company, Ginkgo Bioworks. Ginkgo's contribution was important, as the design and implementation of the mRNA vaccine used Engineering Biology techniques. With mRNA vaccines it was possible to design them to specifically mimic the spike protein of SARS‐CoV‐2 to teach the body to make copies of the protein. Hence, the body's immune system has a direct template that causes the immune system to react in the presence of the virus. A key point is that because of the direct nature of the design, future iterations of the vaccine design can be made to optimise it for new strains of the virus (the so‐called plug‐and‐play approach).

It is now becoming clear that a distributed model for the manufacture of vaccines, whilst not straightforward, has advantages. The distributed model differs in several ways from the traditional vaccine production methods—and there is a range of different methods, based in Engineering Biology, for the production of vaccines that are not based on traditional methods. One of the number of examples described in a recent Financial Times article [34] is the Medicago/GSK methodology that involves the manufacture of large quantities of the vaccine in the leaves of tobacco plants. In the case of the mRNA vaccines, it is reported that the number of doses that can be obtained from a given volume of ‘broth’ is significantly greater than for traditional vaccines. This means that, in principle, it should be possible to produce vaccines at a local level in relatively small quantities (but providing large numbers of doses). This would immediately minimise the cold chain problem. The second important point is that it may be possible to design production facilities that are significantly based on the Biofoundry technology that is now being used in Engineering Biology. With these provisos, it might be possible to install such a production facility in, for example, a university or research facility in Africa and to control it remotely across the Internet, with minimal human intervention. Such a distributed model would be attractive to governments around the world and to COVAX‐WHO. The global pandemic of COVID‐19 has produced major economic disruption. This is not something that the international governmental community will want to repeat.

But, Engineering Biology is platform technology that has a much wider range of applications than biomedicine. It is particularly important in addressing the green industrial revolution and zero carbon targets proposed by many governments. The development of the BioEconomy and economic sustainability are seen as vital from this point of view; also, in terms of simply growing the economy. Several countries, in different parts of the world, have now produced strategic roadmaps for the development of Engineering Biology—and have addressed the importance of the field in growing their BioEconomies.

As described earlier in this paper, in the United Kingdom there have been three important strategic documents published (starting in 2009 with The Royal Academy of Engineering report on Synthetic Biology and the two later UK Roadmaps on Synthetic Biology [2, 4, 18] defined the strategy that has now resulted in the establishment of a nationwide infrastructure comprising seven major basic research centres and a national centre for industrial translation. These centres, together with several other centres and around 180 start‐ups and SMEs, represent a strong base for the development of the UK's BioEconomy.

In the United Kingdom, we are now at an important crossroad where the achievements to date can be lost by inadequate strategy for the next stages of the development of Engineering Biology. Unlike the situation in 2009, when the field was in its infancy (at least as far as industrial translation was concerned), today there are established strategies (internationally) for the next stages of the development of the field. What is clear is that, by far the best approach to the development of future strategy is to involve a wide a range of practitioners/experts from academia, industry and business in the development of the strategy at all stages. The classic example of this approach is the development of the Roadmap for Engineering Biology by the Engineering Biology Research Consortium (EBRC) in the United States. The EBRC encouraged as many experts in the field as were willing to participate to work on the development of all stages of the Roadmap and its implementation.

This process of total transparency is an outstanding model of how to develop, what is, in effect, a national strategy for the development of the field in the post‐COVID era—and, concomitantly, the development of the green industrial revolution and low‐carbon economies. The wrong approach is for government agencies to develop a national strategy and to only involve the full, wide‐ranging participation of experts in the field post hoc. In the case of the EBRC roadmap, the aim was and is set out in the mission statement.

“It is intended to provide researchers and other stakeholders across engineering biology disciplines and industries with a consolidated view of challenges and opportunities in the near and long term. The framework of the Roadmap focusses on the development and advancement of tools and technologies in engineering biology and their potential applications and impact.” https://ebrc.org/programs/research‐roadmap‐program/

The strategy for the EBRC Roadmap was and is to engage groups of experts in various aspects of the field to develop the strategy for their area of expertise. The leader(s) of each group are members of an overall strategic committee and, again, they provide full transparency back to the individual groups of experts. The importance of this approach is twofold. First, each of the subgroups work in their areas of expertise and comprise experts from, for example, academia, industry and business—as appropriate. Second, even though the original Roadmap was published in 2019, it is a living document on the web and is continuously updated. In addition, the aspects of the field that require more emphasis (e.g. biosecurity) are enhanced, when appropriate.

## 10 IN SUMMARY

The Green Industrial Revolution and Zero Carbon, coupled to the development of the BioEconomy, are vital for the future of the planet. The field of Engineering Biology will play a key role in these developments—but only if the correct strategy for its future development is developed by a wide range of experts from academia, industry and business working in unison.

In the United Kingdom, the public investment in the field of Engineering Biology (approximately £350 million) in the years 2013–2020 attracted around six times the amount of investment from the private sector. It is clear from international evidence that public sector investment is essential to continue to attract increasing levels of private sector investment. Hence, for the United Kingdom to develop its industrial base in Engineering Biology effectively, at least three components are needed:

(i) Adequate, continued, stable long‐term public investment to properly support and expand the country's excellent Engineering Biology infrastructure
(ii) Implementation of the type of strategy that has been developed by the EBRC—that includes the continued commitment of a wide range of experts in the field from academia, industry, governmental agencies and NGOs
(iii) The development of a specially‐trained expert workforce

If we can achieve this, then the field of Engineering Biology can have a major impact on both the development of the BioEconomy in many areas (including healthcare) and climate change.

## CONFLICT OF INTEREST

No conflicts of interest.

## PERMISSION TO REPRODUCE MATERIALS FROM OTHER SOURCES

Permissions have been given to reproduce materials from other sources.

## Data Availability

Data sharing not applicable to this article as no datasets were generated or analysed during the current study.

## Footnotes

^1^
Internationally, the terms Engineering Biology and Synthetic Biology are often used interchangeably. For the purpose of simplicity, the term Engineering Biology will be used throughout this work except where either term is specifically used by a particular source.

^2^
SBLC—now renamed the EBLC (Engineering Biology Leadership Council).

^3^
BEIS—The UK Government's Department of Business, Energy and Industrial Strategy.
